# Supplementary material for: Role of mTOR through Autophagy in Esophageal Cancer Stemness
Source: Cancers (Basel). 2022 Apr 1;14(7):1806. doi: 10.3390/cancers14071806 (PMC9040713; doi:10.3390/cancers14071806)
Supplement: Supplementary file 1 [file cancers-14-01806-s001.zip › Supplementary data Cancers 1605530-20220330.pdf]

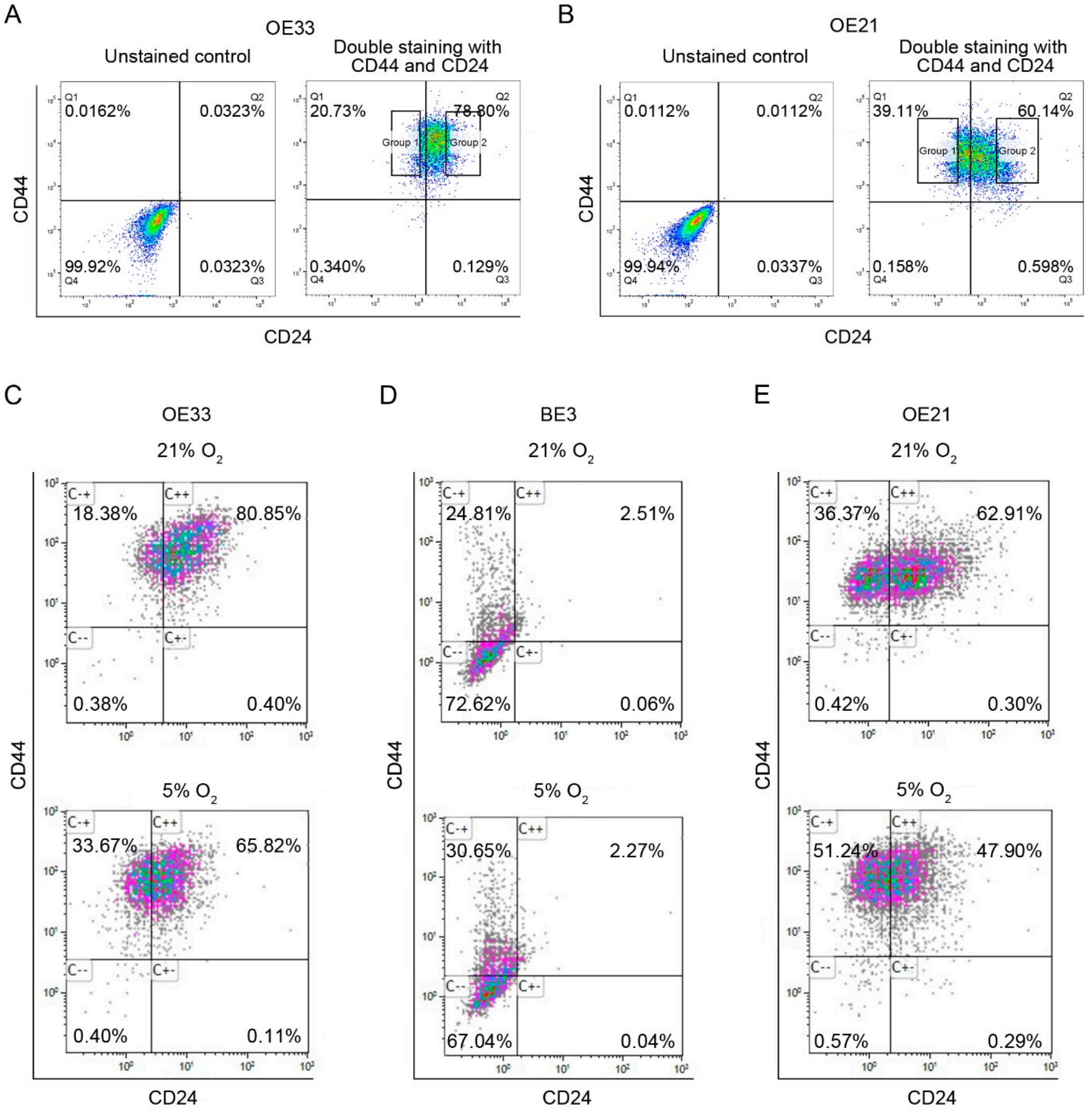

Supplementary Figure S1: Gating strategies of the FACS experiment. (A-B) The left 3-15% proportion of CD44$^+$/CD24$^-$ cells and the right 3-15% populations proportion of CD44$^+$/CD24$^+$ cells were sorted for the western blot. **(C-E)** Representative FACS plots of the percentage of CD44$^+$/CD24$^-$ cells in OE33, BE3 and OE21 cultured under normoxic conditions and low oxygen (5% O2, 48h) environment.

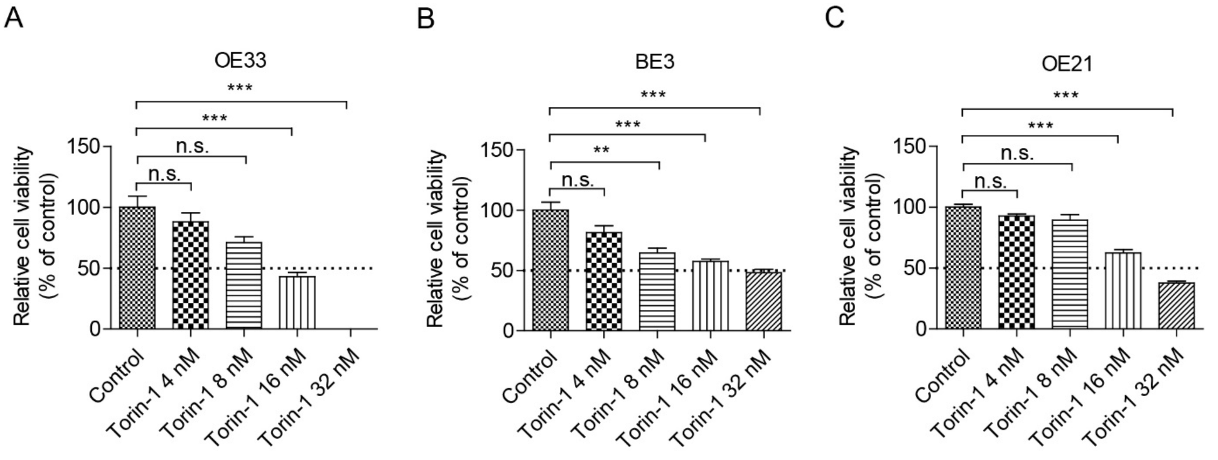

Supplementary Figure S2: Viability of cells treated with Torin-1 (4, 8, 16, or 32 nM) or vehicle solution for 48 h.

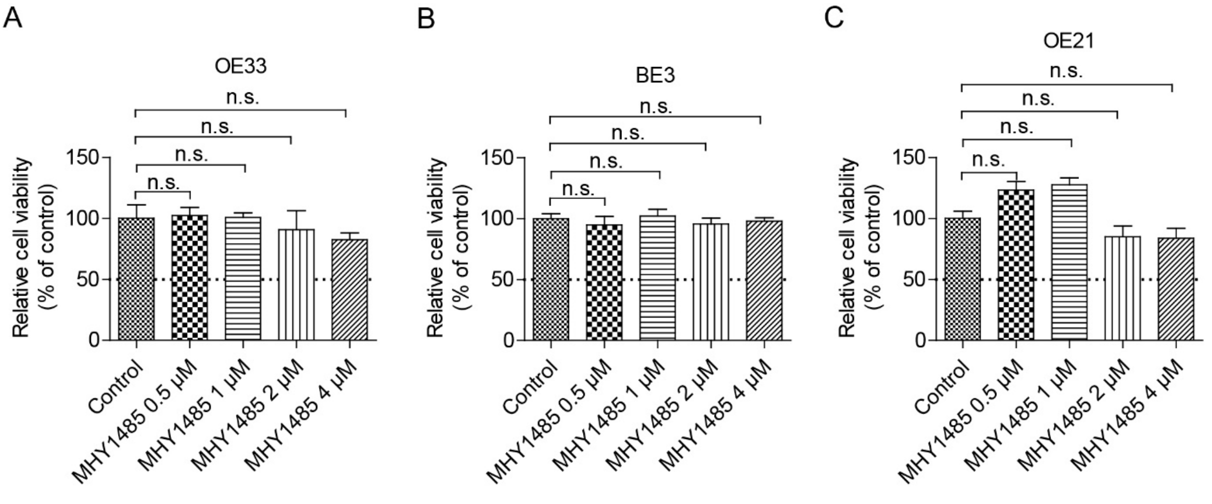

Supplementary Figure S3: Viability of cells treated with MHY1485 (0.5, 1, 2, or 4µM) or vehicle solution for 48 h.

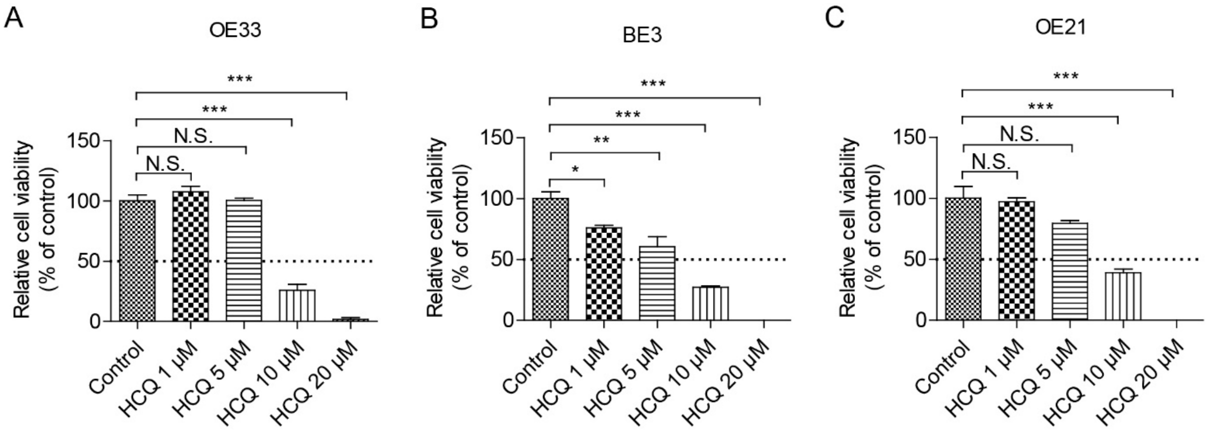

Supplementary Figure S4: Viability of cells treated with HCQ (1, 5, 10, or 20μM) or vehicle solution for 48 h.

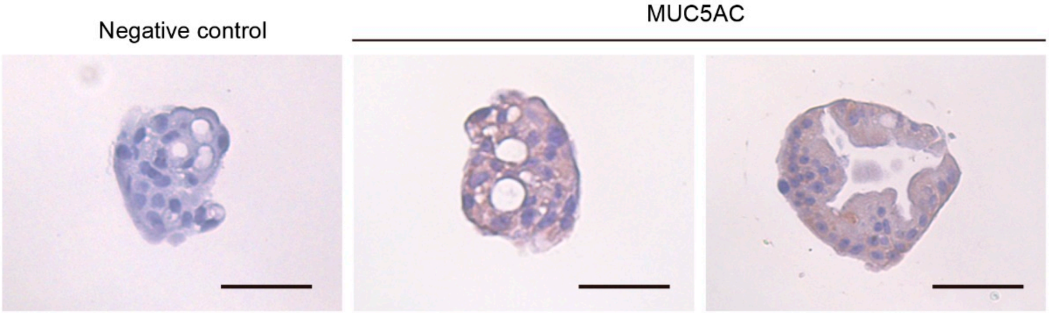

Supplementary Figure S5: Representative images of positive cytoplasmic staining for EAC marker MUC5AC in ec-PDOs. Scale bar = 100 µm.

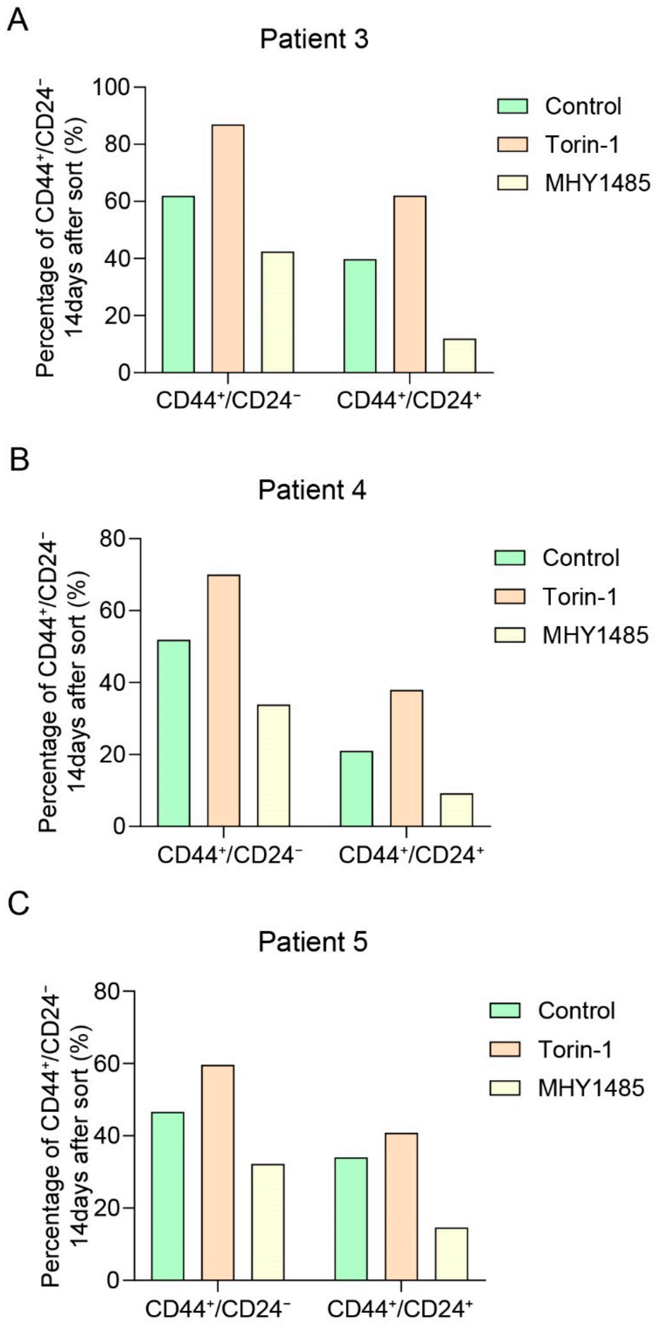

Supplementary Figure S6: The percentage of $CD44^+/CD24^-$ in patient material after sort and treated with Torin-1 (10nM, 14 days) or MHY1485 (1µM, 14 days).
