# Supplementary material for: Role of mTOR through Autophagy in Esophageal Cancer Stemness
Source: Cancers (Basel). 2022 Apr 1;14(7):1806. doi: 10.3390/cancers14071806 (PMC9040713; doi:10.3390/cancers14071806)
Supplement: Supplementary file 1 [file cancers-14-01806-s001.zip › Supplementary material-Original Western blot.pdf]

Supplementary Materials

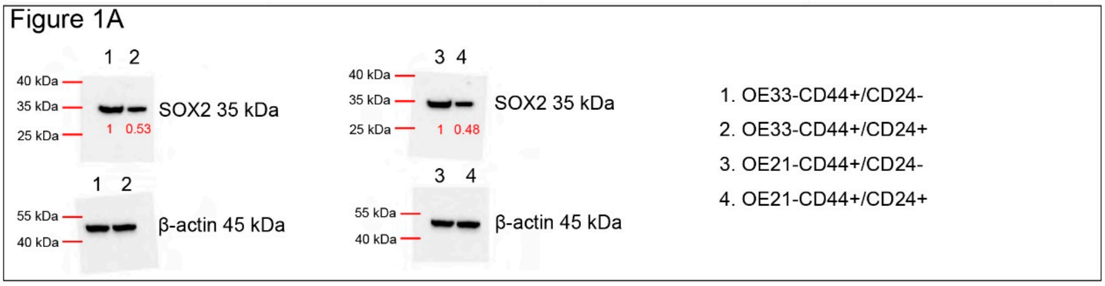

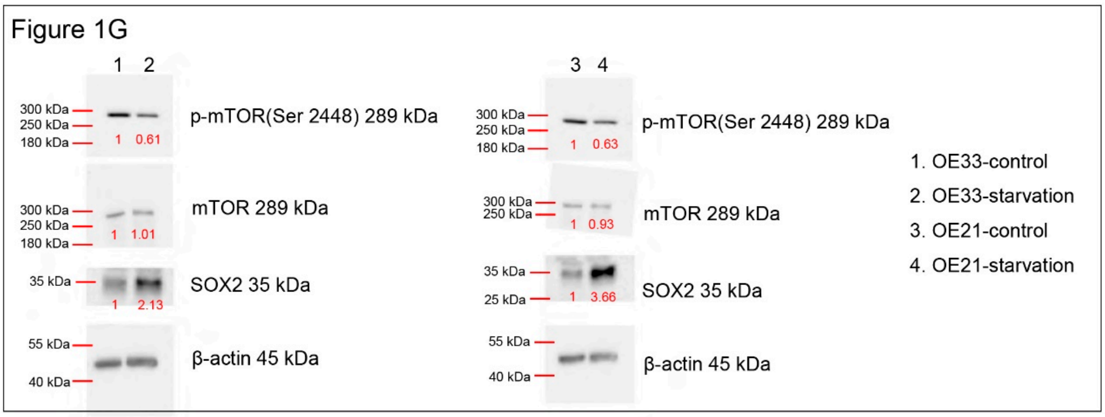

Original western blot images of Figure 1.

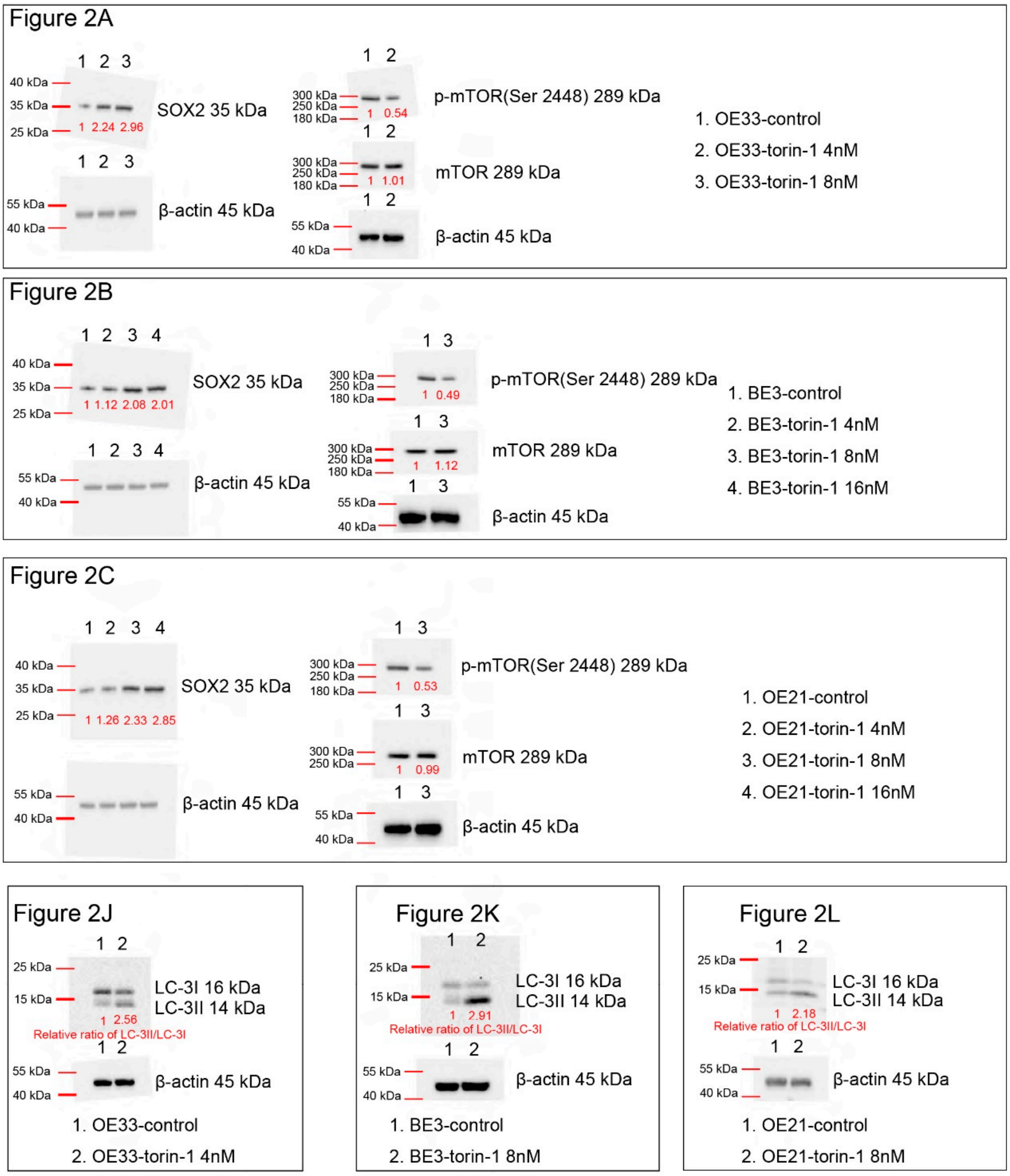

Original western blot images of Figure 2.

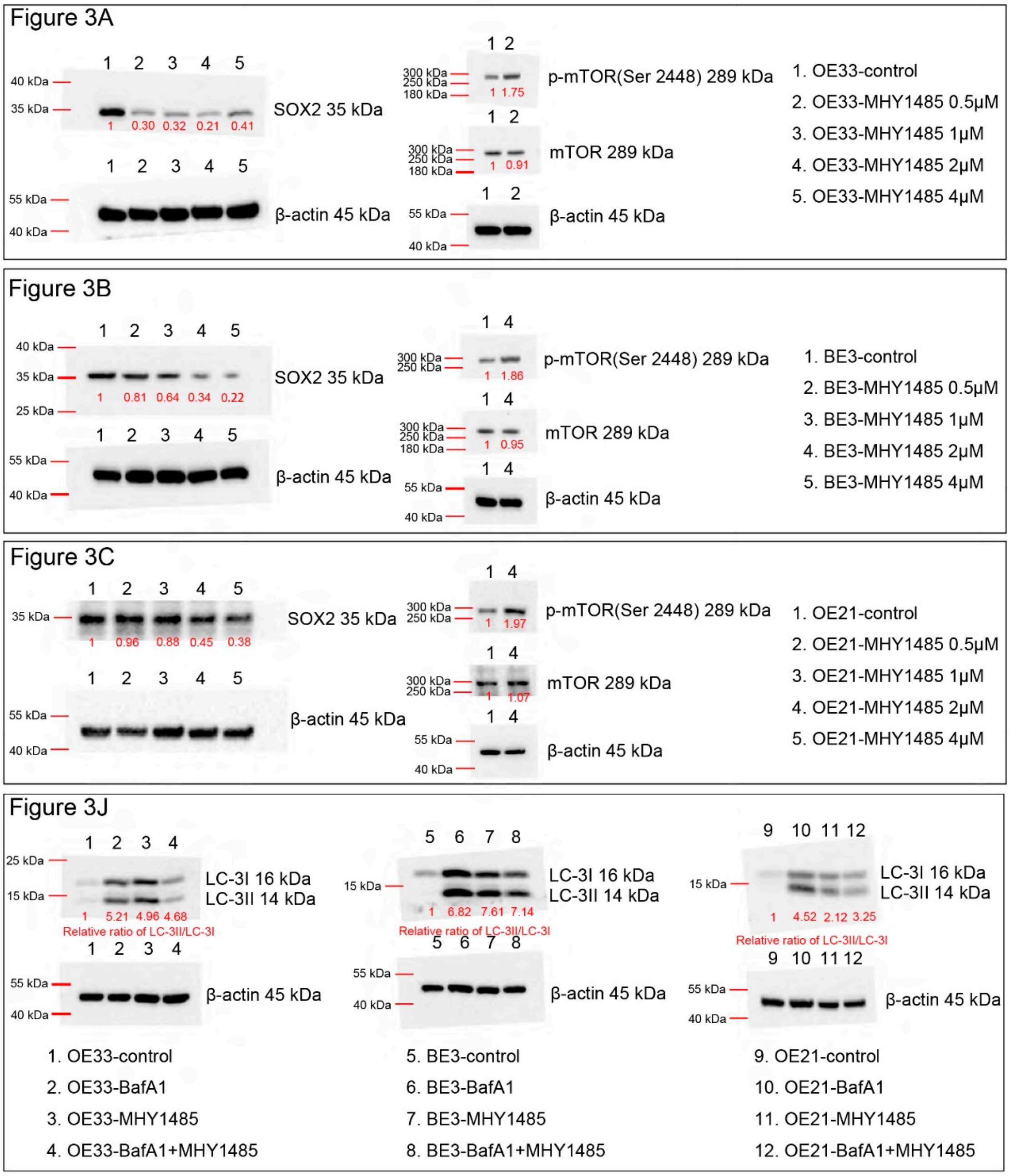

Original western blot images of Figure 3.

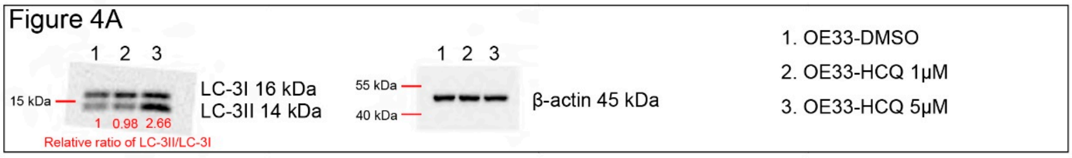

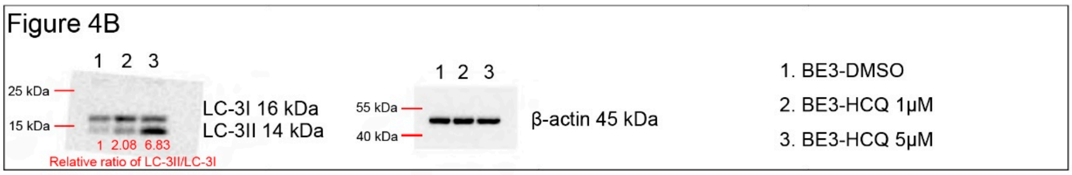

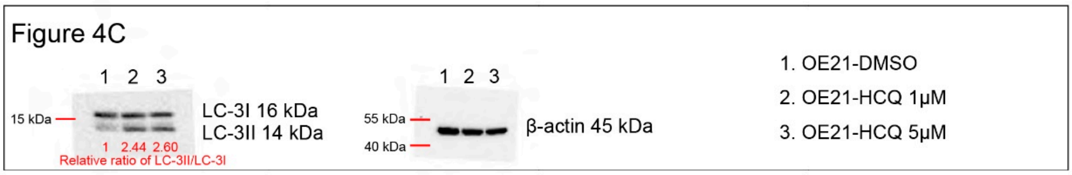

Original western blot images of Figure 4.
